# Supplementary material for: The interactome of intact mitochondria by cross-linking mass spectrometry provides evidence for coexisting respiratory supercomplexes
Source: Mol Cell Proteomics. 2017 Dec 8;17(2):216–32. doi: 10.1074/mcp.RA117.000470 (PMC5795388; doi:10.1074/mcp.RA117.000470)
Supplement: Supplemental Data [file supp_RA117.000470_133922_0_supp_23978_fzffwf.zip › spectra_annotation/mito_DR_spectra_annotation/72-1-5-1-11-1.pdf]

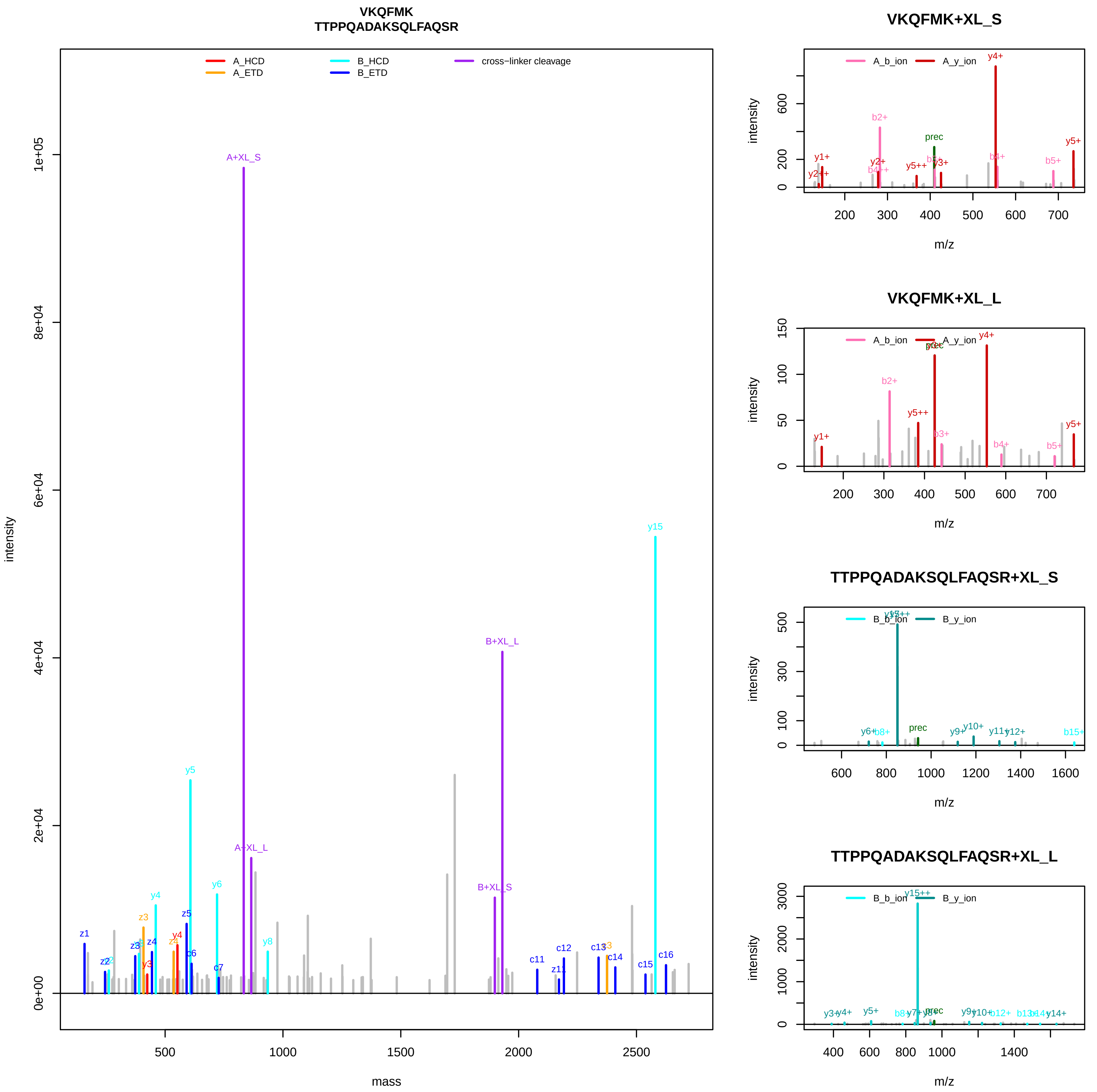

VKQFMK
TTPPQADAKSQLFAQSR
A_HCD
A_ETD
B_HCD
B_ETD
cross−linker cleavage
A+XL_S
A+XL_L
B+XL_L
B+XL_S
y15
y5
y6
y4
y8
z1
z2
z3
z4
z5
c6
c7
y3
y4
c11
z11
c12
c13
c14
c15
c16
intensity
mass
0e+00
2e+04
4e+04
6e+04
8e+04
1e+05
500
1000
1500
2000
2500
VKQFMK+XL_S
A_b_ion
A_y_ion
y4+
b2+
prec
y5+
y1+
y2++
y2+
y5++
y3+
b4+
b5+
m/z
VKQFMK+XL_L
TTPPQADAKSQLFAQSR+XL_S
B_b_ion
B_y_ion
y15++
y6+
b8+
y9+
y10+
y11+
y12+
b15+
TTPPQADAKSQLFAQSR+XL_L
